# Early‐stage randomised controlled trial of therapist‐supported online cognitive therapy for post‐traumatic stress disorder in young people

**DOI:** 10.1111/jcpp.14124

**Published:** 2025-02-06

**Authors:** Patrick Smith, Anke Ehlers, Ewan Carr, David M. Clark, Tim Dalgleish, Gordon Forbes, Kimberley Goldsmith, Helena Griffiths, Monica Gupta, Dorothy King, Sarah Miles, Dominic T. Plant, Anne Smith, Jess Steward, William Yule, Richard Meiser‐Stedman

**Affiliations:** ^1^ Department of Psychology Institute of Psychiatry, Psychology and Neuroscience, King's College London London UK; ^2^ South London & Maudsley NHS Foundation Trust London UK; ^3^ Department of Experimental Psychology University of Oxford Oxford UK; ^4^ Department of Biostatistics and Health Informatics Institute of Psychiatry, Psychology and Neuroscience, King's College London London UK; ^5^ Medical Research Council Cognition and Brain Sciences Unit University of Cambridge Cambridge UK; ^6^ Cambridgeshire and Peterborough NHS Foundation Trust Cambridge UK; ^7^ Department of Clinical Psychology and Psychological Therapies, Norwich Medical School University of East Anglia Norwich UK

**Keywords:** Post‐traumatic stress disorder, adolescence, cognitive therapy, E‐health, Randomised Controlled Trial

## Abstract

**Background:**

Effective face‐to‐face treatments for Post‐Traumatic Stress Disorder (PTSD) are available, but most young people with PTSD do not receive effective treatment. Therapist‐supported online Cognitive Therapy has the potential to improve accessibility of effective treatment. This early‐stage trial gathered data on the feasibility, acceptability, and initial signal of clinical efficacy of a novel online Cognitive Therapy program for young people with PTSD.

**Methods:**

A two‐arm, parallel‐groups, single‐blind, early‐stage feasibility RCT compared online Cognitive Therapy to a waitlList condition. Participants were *N* = 31 adolescents (12–17 years‐old) with a diagnosis of PTSD, randomised in a 1:1 ratio using minimisation. Thresholds for progression to a larger trial were set a priori for recruitment rate, data completeness, and the initial signal of clinical efficacy. The primary clinical outcome was PTSD diagnosis at 16 weeks post‐randomisation. Secondary clinical outcomes were continuous measures of PTSD, depression, and anxiety at 16 weeks; and at 38 weeks in the online Cognitive Therapy arm.

**Results:**

All pre‐determined feasibility thresholds for progression to a larger trial were met. We recruited to target at a rate of 1–2 participants/month. No patient dropped out of therapy; 94% of all participants were retained at 16 weeks. At 16‐weeks, the intention‐to‐treat (ITT) effect adjusted odds ratio was 0.20 (95% CI, 0.02, 1.42), indicating that the odds of meeting PTSD caseness after online therapy were 80% lower than after the waitlist (10/16 participants met PTSD caseness after therapy compared to 11/13 after WL). Effect‐size estimates for all secondary clinical outcomes were large‐moderate; improvements were sustained 38 weeks after online Cognitive Therapy.

**Conclusions:**

Therapist‐supported online Cognitive Therapy for PTSD is acceptable to young people and has potential for meaningful and sustained clinical effects. A larger trial appears feasible to deliver. Further work is needed to refine the intervention and its delivery and to evaluate it in a larger confirmatory trial.

## Introduction

Post‐Traumatic Stress Disorder (PTSD) in young people is highly distressing, associated with marked impairments in functioning, and can persist for years (Yule et al., 2000) or decades (Morgan, Scourfield, Williams, Jasper, & Lewis, 2003) if not treated. Trauma‐Focused Cognitive Behavioural Therapies (TF‐CBTs) are highly effective in reducing PTSD symptoms when delivered in person (Gutermann et al., 2016; Mavranezouli et al., 2020; Morina, Koerssen, & Pollet, 2016) and are recommended in international practice guidelines as the first‐line treatment for PTSD in young people (Forbes, Bisson, Monson, & Berliner, 2019; NICE, 2018; Phelps et al., 2022).

Unfortunately, most young people with PTSD do not receive an evidence‐based therapy (Lewis et al., 2019). This is likely due to multiple interacting factors, including under‐capacity and long waiting times in child and adolescent mental health services, the burden and inconvenience to young people and families in attending in‐person appointments, and possible stigma around help seeking. Wider availability of effective treatments for PTSD in young people is urgently needed.

Online therapy has the potential to address this need. Digital health interventions are broadly acceptable to young people and can be clinically helpful in treating depression and anxiety in adolescents (Christ et al., 2020; Hollis et al., 2017). However, digital interventions for Post Traumatic Stress Symptom (PTSS) in young people are relatively few and vary widely in their content, delivery, and intended use (Schulte, Harrer, Sachser, Weiss, & Zarski, 2024). Most digital interventions for PTSS in young people are CBT‐based, but some comprise psychoeducation alone or are meditation‐based. Most are delivered in a modular format via the internet, but gamified interventions via a smartphone app also exist. Interventions are intended for use as universal or targeted prevention strategies or for treatment of PTSS. A recent meta‐analysis (Schulte et al., 2024) of 5 controlled evaluations (Cox, Kenardy, & Hendrikz, 2009; Kassam‐Adams et al., 2016; Ruggiero et al., 2015; Schuurmans, Nijhof, Scholte, Popma, & Otten, 2020; van Rosmalen‐Nooijens, Lo Fo Wong, Prins, & Lagro‐Janssen, 2017) reported a small within‐group effect size on PTSS (Hedges *g* = −0.39, 95% CI −0.11 to −0.67) and negligible between‐group effects relative to a variety of comparators (including Wait List) in 3 studies (Hedges *g* = 0.04, 95% CI 0.06 to −.52). Of the 5 RCTs to date, only one required elevated PTSS for participant inclusion, and none required PTSD diagnosis for inclusion. To our knowledge, no study has yet reported on the development and evaluation of an online course of therapy for treatment‐seeking young people with a diagnosis of PTSD.

Cognitive Therapy for PTSD in Young People (CT‐PTSD‐YP) is a form of TF‐CBT developed by our group. The treatment is theory‐driven (Ehlers & Clark, 2000), developed from adult protocols (Ehlers, Clark, Hackmann, McManus, & Fennell, 2005), manualised for use with young people (Smith, Perrin, Yule, & Clark, 2010), and is delivered over 10–12 individual face‐to‐face sessions. Three published RCTs found that when delivered face‐to‐face to children and young people, CT‐PTSD‐YP is acceptable and efficacious (Hitchcock et al., 2022; Meiser‐Stedman et al., 2017; Smith et al., 2007). A therapist‐assisted internet‐delivered version of CT‐PTSD for adults showed very large symptom improvement of PTSD, depression, and anxiety and superiority to internet‐delivered stress management in reducing symptoms in a recent trial (Ehlers et al., 2023).

We have developed an online version of CT‐PTSD‐YP (see Appendix S1 for further detail). We used co‐design principles to involve young people in developing the therapy App and website (Bevan Jones et al., 2020), and we designed for remote support from a therapist to facilitate the active engagement of young people in therapy (Hollis et al., 2017). Before the current study, the acceptability to young people of this online therapy was unknown, and the feasibility of testing its efficacy in a confirmatory RCT was uncertain.

Our aim in this early‐stage trial was therefore to gather data on the feasibility, acceptability, and initial signal of clinical effect of therapist‐supported internet‐delivered CT‐PTSD‐YP (hereafter iCT‐PTSD‐YP; Smith et al., 2022) to inform the viability, design, and size of a future confirmatory trial. Our longer‐term intention is to determine whether this approach will help to reduce the treatment gap for young people with PTSD by making efficacious therapy more widely available.

## Method

### Trial ethics and registration

The trial was approved by a UK Health Research Authority (HRA) Research Ethics Committee (19/LO/1354) and prospectively registered (including the study protocol and statistical analysis plan) with the ISRCTN (registry ID number 16876240; https://doi.org/10.1186/ISRCTN16876240). Written informed consent was obtained from participants for those aged 16 years and older and from parents for those younger than 16 years.

### Objectives

The primary objective was to gather data on the feasibility, acceptability, adherence, retention, and delivery of iCT‐PTSD‐YP. The secondary objective was to provide exploratory point estimates of the superiority comparison effect sizes (and their confidence intervals) of iCT‐PTSD‐YP relative to a wait‐list condition on symptoms of PTSD, anxiety, and depression.

### Design

We did a two‐arm, parallel groups, single‐blind (outcome assessor), early‐stage RCT comparing iCT‐PTSD‐YP with a waitlist (WL) condition for young people aged 12–17 years with a diagnosis of PTSD. The primary feasibility and acceptability indices and thresholds were set a priori. The primary clinical outcome was PTSD diagnosis (ascertained using the Clinician Administered PTSD Scale for DSM‐5: Child and Adolescent version, CAPS‐CA‐5; Pynoos et al., 2015) at 16 weeks post‐randomisation; secondary clinical outcomes were continuous measures of post‐traumatic stress symptoms, depression, and anxiety at 16 weeks and the same measures at 38 weeks follow‐up in the iCT‐PTSD‐YP arm only (see Table S1 for measures and timeline).

### Participants

Inclusion criteria for young people were: 12–17 years old; main presenting problem is PTSD diagnosis ascertained using the CAPS‐CA‐5 (Pynoos et al., 2015); PTSD symptoms relate to a single trauma; English language skills sufficient to allow therapy without an interpreter and independent use of iCT‐PTSD‐YP; access to a smartphone and a larger device (laptop, desktop computer, tablet) with internet access; and access to a safe and confidential space to engage in iCT‐PTSD‐YP. Exclusion criteria were: brain damage, intellectual disability, pervasive developmental disorder or neurodevelopmental disorder; other psychiatric diagnosis that requires treatment before PTSD; moderate to high risk to self; ongoing trauma‐related threat; started or changed treatment with psychotropic medication within the last 2 months; currently receiving another psychological treatment; or previously received TF‐CBT in relation to the same traumatic event. Inclusion criteria for carers were: parent or carer of a young person who meets all the inclusion criteria and none of the exclusion criteria above; English language skills sufficient to allow participation without an interpreter and to allow independent use of iCT‐PTSD‐YP; and access to a smartphone or larger device with internet access. Young people were eligible to participate without the involvement of their carer in treatment. Referrals were sought from UK National Health Service (NHS) Child and Adolescent Mental Health Services (CAMHS) in London and Southeast England and from secondary schools in the same regions. We had also planned to carry out screening in schools using the Children's Revised Impact of Event Scale (CRIES‐8; Perrin, Meiser‐Stedman, & Smith, 2005), but this was not possible due to the COVID‐19 pandemic. We aimed to recruit *N* = 14 per arm at post‐treatment (aimed to randomise *N* = 34 to allow for ~20% drop out) to gather meaningful data on feasibility and acceptability. This sample size does not provide sufficient statistical power for definitive between‐group comparisons.

### Interventions

#### iCT‐PTSD‐YP

Internet‐delivered Cognitive Therapy for PTSD for young people (iCT‐PTSD‐YP) comprises therapist‐supported online delivery of all components from our published manual of face‐to‐face CT‐PTSD for young people (Smith et al., 2010). Please see Appendix S1. Treatment components are delivered online via a smartphone App and/or website in a modular format. Module content was informed by iCT‐PTSD for adults (Ehlers et al., 2023), and modules were co‐designed with input from young people. Eleven core modules are intended for all young people (Psychoeducation about PTSD, Reclaiming life, Understanding PTSD, Developing a trauma narrative, Identifying hotspots, Updating the narrative, Working with triggers, Overcoming sense of danger, Visiting the site virtually, Viewing the site in person, Developing a blueprint). Eleven additional optional modules can be used if required (Anger, Grief, Shame, Guilt, Self‐criticism, Rumination, Working with images, Working with physical difference, Relaxation, Sleep, and Panic). Core and optional modules are released sequentially by the therapist according to individual need. Once released, modules can be used for independent self‐study. Modules are interactive and include text, illustrations, audio case examples, animations, and videos. The App includes a messaging function for young people to communicate with their therapist. Treatment length was planned for 12 weeks to mirror our face‐to‐face CT‐PTSD protocol, but additional treatment before the 16‐week primary endpoint was permitted if needed.

Parents and carers were given separate credentials to access a carer version of the App. The carer version comprises eight modules that provide information about PTSD and therapy, including advice about how carers can help young people.

Therapists contact young people and carers via phone or videoconferencing at least once a week for the duration of therapy. Therapists can log onto the site to view young people's progress, including their text input and questionnaire responses, and therapists can add written responses or suggestions to the young person's modules, which young people can view online. Therapists in this study were doctoral‐level clinical psychologists or CBT therapists (DK, SM, AS) who had received training in face‐to‐face CT‐PTSD‐YP and the use of the iCT‐PTSD‐YP App and who received weekly clinical supervision from a consultant clinical psychologist (PS, WY).

#### Wait list

Participants allocated to WL did not receive therapy during the waiting period. After assessment at 16 weeks, participants who remained symptomatic were offered iCT‐PTSD‐YP.

### Procedure

#### Randomisation

Participants were randomised independently using a bespoke online randomisation system developed and maintained by the King's College London Clinical Trials Unit, using minimisation with a random component to allocate participants in a 1:1 ratio to iCT‐PTSD‐YP or WL. Minimisation factors were sex and baseline PTSD symptom severity assessed by the Child Post‐traumatic Stress Scale (CPSS‐5; Foa, Asnaani, Zang, Capaldi, & Yen, 2018; (low: <51, high: ≥51)). Research assistants entered the minimisation factors into the online system.

#### Assessment schedule

All participants were assessed pre‐randomisation (baseline), at 6 weeks (mid‐treatment/wait), and 16 weeks (post‐treatment/wait) post‐randomisation. Participants in iCT‐PTSD‐YP were also assessed at 38 weeks post‐randomisation (follow‐up). See Table S1.

#### Outcome measures

##### Feasibility and acceptability outcomes

We planned to count:

1. The number of young people referred to the trial in total and according to referral route;
2. The number of young people screened in schools (using the Children's Revised Impact of Event Scale (CRIES‐8; Perrin et al., 2005)), and the proportion of those who proceeded to a phone call with the family;
3. The number and proportion of young people in schools who scored above the cut‐off on a validated screening questionnaire (CRIES‐8; Perrin et al., 2005) relative to the number of young people screened in schools;
4. The number and proportion of young people in schools who scored above cut‐off on the screening questionnaire but declined further participation with the trial relative to those scoring above cut‐off;
5. The number and proportion of young people in schools who scored above the cut‐off on the screening and consented to further assessment but were deemed ineligible at baseline assessment relative to those deemed eligible at baseline assessment;
6. The number of assessment appointments offered to participants;
7. The number and proportion of assessment appointments attended by participants, relative to the number of appointments offered, reported by referral source;
8. Reasons for not attending assessment appointments, reported by referral source;
9. The number and proportion of young people who, at baseline assessment, consented to participate in the trial, relative to the number who attended assessment, with reasons for not consenting if known;
10. The number and proportion of young people eligible for the trial after baseline assessment, relative to the number of baseline assessments completed;
11. The number and proportion of young people randomised and the proportion of consented young people who were randomised relative to the number who consented;
12. Reasons for withdrawing from the trial, if known; and
13. The number retained in the study at 16 weeks (post‐treatment) and at 38 weeks (follow‐up), and the proportions of those who started treatment who were retained.

##### Adherence outcomes

For participants allocated to iCT‐PTSD‐YP, we counted:

1. The number of times logged into the program per week and in total;
2. Time spent logged in per week and in total;
3. The number of modules completed in total and according to the device used;
4. The number of therapist phone calls attended per week and in total, and the number of missed phone appointments;
5. Time spent on phone calls per week and in total;
6. The number of messages to/from therapist per week and in total;
7. The number and proportion of young people who start treatment;
8. The number of weeks of therapy completed; and
9. The reasons for dropping out of treatment, if known.

##### Clinical outcomes

The primary clinical outcome was a DSM‐5 PTSD diagnosis ascertained using the Clinician Administered PTSD Scale for DSM‐5: Child and Adolescent version (CAPS‐CA‐5; Pynoos et al., 2015), administered remotely using videoconferencing by trained postgraduate or post‐doctorate psychologists blind to treatment allocation. Seventeen randomly chosen interviews were double‐rated to assess reliability. For diagnosis, Cohen's kappa was 0.68 (“substantial agreement”); for symptom severity, the intraclass correlation coefficient was 0.98 (“excellent agreement”). Secondary clinical outcomes reported by young people were: PTSD symptom severity measured by the CAPS‐CA‐5, the Child PTSD Symptom Scale for DSM‐5 (CPSS‐5; Foa et al., 2018), and the Children's Revised Impact of Event Scale (CRIES‐8; Perrin et al., 2005); and symptoms of depression and anxiety on the Revised Children's Anxiety and Depression Scale (RCADS‐C; Chorpita, Ebesutani, & Spence, 2014). Secondary clinical outcomes reported by carers were the young person's symptoms of depression and anxiety using the Revised Children's Anxiety and Depression Scale–Parent version (RCADS‐P; Chorpita et al., 2014) and the young person's emotional and behavioural difficulties using the Strength & Difficulties Questionnaire–parent version (SDQ‐P; Goodman, 2001).

#### Mechanism measures

We included three measures at mid‐treatment/wait to test theoretically derived potential mechanisms of action (mediators) of iCT‐PTSD‐YP on PTSD symptoms: (1) the Child Post Traumatic Cognitions Inventory (CPTCI; McKinnon et al., 2016); (2) the Trauma Memory Quality Questionnaire (TMQQ; Meiser‐Stedman, Smith, Yule, & Dalgleish, 2007); and (3) items from the Trauma Related Rumination Questionnaire (Meiser‐Stedman et al., 2014).

#### Adverse events

Adverse events were broadly defined as any untoward occurrence in a trial participant during the study period. Serious adverse events were defined above as any adverse event that resulted in death, was life‐threatening, required or prolonged hospitalisation, or resulted in persistent or significant disability or incapacity. Adverse events were assessed systematically via interview at mid‐treatment and 16 weeks in both arms and at 38 weeks in iCT‐PTSD‐YP. Adverse events were also monitored during clinical contact for those allocated to iCT‐PTSD‐YP.

#### Statistical analyses

Feasibility and adherence metrics were described using appropriate summary statistics (e.g., mean and standard deviation or median and IQR for continuous outcomes; number and percentage for categorical outcomes). Confidence intervals for the recruitment rate were calculated using exact 95% Poisson confidence intervals (Ulm, 1990). Clinical outcomes were described by time point, arm, and overall, using appropriate summary statistics.

The primary analyses were conducted using Intention to Treat (ITT) principles. We estimated the iCT‐PTSD‐YP versus WL treatment effect at 16 weeks, with the appropriate 95% confidence interval. For the primary clinical outcome (remission from PTSD caseness at 16 weeks post‐randomisation), we calculated the iCT‐PTSD‐YP versus WL odds ratio using logistic regression, with trial arm and the minimisation variables as covariates. For secondary outcomes, we estimated the iCT‐PTSD‐YP versus WL mean differences at 16 weeks post‐randomisation using linear regression, with trial arm, baseline outcome score, and minimisation variables as covariates. Cohen's d effect sizes were calculated for these outcomes by dividing the estimated mean difference from the linear regression models by the pooled baseline standard deviation across the whole trial population.

We conducted secondary analyses of the primary outcome at 16 weeks using two per‐protocol populations: “Minimum therapy needed to achieve benefit” (participants who completed a minimum set of 6 (of 11) core therapy modules) and “Broader population” (additionally completed a module on triggers; see Appendix S2 for details). Where between‐arm analyses are presented, they are underpowered and should be considered preliminary and not be interpreted as evidence of the effectiveness of the intervention.

We report numbers in each arm achieving reliable change in post‐traumatic stress symptom severity on the CRIES‐8 and CPSS‐5. The reliable change index (RCI) is calculated using normative data on variance and reliability (Jacobson, Follette, & Revenstorf, 1984). For the CRIES‐8, we used the published RCI of 11.92, derived from a national UK dataset (Child Outcome Research Consortium [CORC], 2016). For the CPSS‐5, we calculated an RCI of 14.87 based on published normative data (CPSS SD = 18.97; reliability = 0.92; Foa et al., 2018).

We conducted an exploratory mediation analysis to estimate the pathways between treatment allocation (iCT‐PTSD‐YP vs. WL), the putative mediators, and the primary outcomes and selected secondary outcomes using separate linear and logistic regression models. We estimated indirect effects using the regression‐based method of Valeri and van der Weele (2013) implemented in the regmedint R package (Yoshida & Li, 2022). For the primary outcome, we used a modified Poisson outcome model (log link with robust variance; Zou, 2004), given the common binary outcome. For secondary outcomes, we used linear regression models for the outcome (see Appendix S3 for details).

#### Progression criteria

We pre‐specified four progression criteria in our protocol (Smith et al., 2022). Thresholds were set on the basis that meeting them would indicate that a future confirmatory trial is likely to be feasible. These were: >65% of eligible participants consent to take part in this trial; >90% of randomised participants provide outcome data on a PTSD measure; >90% of trial completers provide complete data on a PTSD measure; and Cohen's *d* ≥ 0.8 for iCT‐PTSD‐YP versus WL on the secondary outcome CPSS.

## Results

### Participant flow

**Figure 1:**
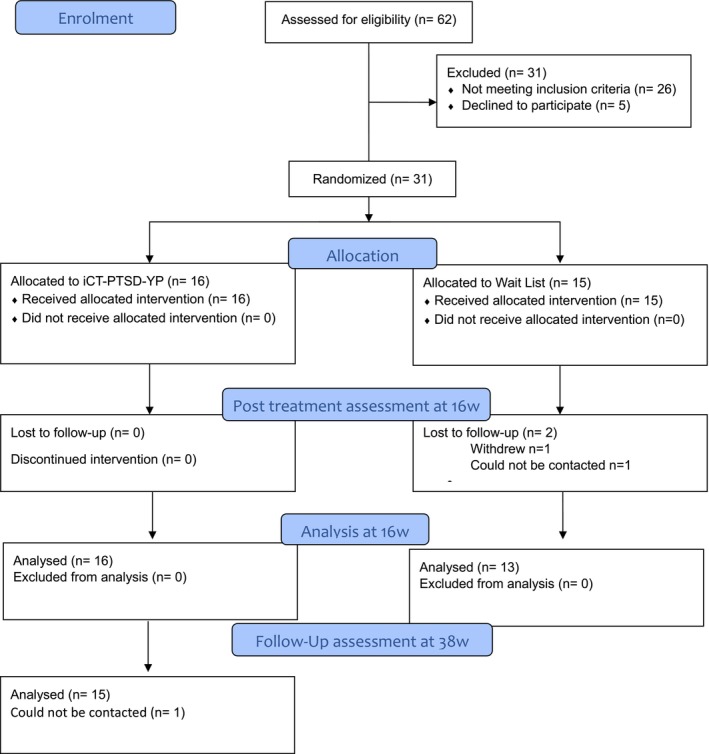
CONSORT diagram

Participant flow is shown in Figure 1 (CONSORT diagram; further details in Figure S1). From March 2020 to October 2021 (19 months), a total of 212 individuals were discussed with referrers, resulting in 162 referrals, of whom 140 were screened via a phone call, and *N* = 62 were assessed. Of those assessed, *N* = 36 (58%) were eligible to participate in the trial, and *N* = 31 (50%) participated and were randomised. A mean of 1.7 (95% CI: 0.9, 2.5) participants were recruited each month.

### Participant characteristics

Demographic and trauma‐related characteristics of participants are presented in Table 1. Participants tended to be female (87%) and of White ethnicity (71%), with an overall median age of 15 years. Most participants were referred to the study via CAMHS (87%). More than half of participants reported severe PTSD symptoms on the CPSS questionnaire at baseline. Most traumas were either sexual assault or rape (42%) or physical assault (39%). The start of treatment was around 1–3 years after the date of trauma. The arms were largely balanced regarding baseline characteristics, although there were some apparent differences in ethnicity and days since trauma.

**Table 1:** Participant characteristics

	Statistic	iCT‐PTSD‐YP	Wait list	Overall
		*n* = 16	*n* = 15	*n* = 31
Age at baseline	Median (IQR)	15 (15, 16)	15 (15, 16)	15 (15, 16)
Gender	*N* (%)			
Male		2 (12)	2 (13)	4 (13)
Female		14 (88)	13 (87)	27 (87)
Prefer not to say		0 (0)	0 (0)	0 (0)
Ethnicity	*N* (%)			
White		10 (62)	12 (80)	22 (71)
Asian or Asian British		0 (0)	1 (7)	1 (3)
Black or Black British		4 (25)	2 (13)	6 (19)
Mixed background		2 (12)	0 (0)	2 (6)
Other ethnicity		0 (0)	0 (0)	0 (0)
Referral source	*N* (%)			
CAMHS		14 (88)	13 (87)	27 (87)
School		1 (6)	0 (0)	1 (3)
Self referral		1 (6)	2 (13)	3 (10)
High symptom severity^a^	*N* (%)	9 (56)	8 (53)	17 (55)
Type of trauma	*N* (%)			
Sexual assault or rape		7 (44)	6 (40)	13 (42)
Physical assault		6 (38)	6 (40)	12 (39)
Transport accident		1 (6)	1 (7)	2 (6)
Medical procedure		0 (0)	1 (7)	1 (3)
Family member ill		1 (6)	0 (0)	1 (3)
Family member dying		1 (6)	1 (7)	2 (6)
Days since trauma^b^	Median (IQR)	738 (598, 1,122)	631 (329, 779)	698 (475, 986)

^a^ Baseline CPSS score ≥ 51.

^b^ Number of days between date of trauma and date of randomisation.

### Feasibility outcomes

#### Referral routes and school screening

Most referrals (78%) and participants (87%) were from CAMHS. We were unable to complete any screenings in schools due to COVID‐19. The numbers of enquiries and assessments via each of the three possible referral routes (school screening, CAMHS, GP, or self‐referral) are shown in Figure S1.

#### Assessments offered and completed

A total of 73 assessment appointments were offered, and 62 (85%) attended. For CAMHS referrals, 52 of 63 (83%) offered appointments were attended. For the other referral routes, all 10 offered appointments were attended. For the 11 who did not attend, the most common reasons were that they did not consent or did not complete online questionnaires before the assessment.

#### Eligibility after assessment

Of 62 young people attending a baseline assessment, 36 (58%) were eligible to participate in the trial. Reasons for ineligibility are detailed in Figure S1. Of the 36 eligible young people, 32 (89%) consented to participate in the trial.

#### Randomisation and retention

Of the 32 young people who consented, 31 (97%) were randomised. One participant reported after giving consent that they had been offered immediate non‐trial psychological intervention, so they chose not to take part in the trial. In the iCT‐PTSD‐YP arm, 16/16 (100%) provided data at 16 weeks; 15/16 (94%) provided information at 38 weeks. In the WL arm, 13/15 (87%) provided data at 16 weeks (1 withdrew between baseline and mid‐WL, no reason given; 1 was lost to follow‐up at 16 weeks).

### Adherence in iCT‐PTSD‐YP

#### Logging on to iCT‐PTSD‐YP

Most participants logged in via phone or computer; very few used a tablet to log in. Participants tended to log in once a week during the intervention period. There were more logins at the beginning (~2/week for the first 3 weeks) than at the end (median 0 for the last 3 weeks) of iCT‐PTSD‐YP. Overall, participants spent around 4 h/week logged in during the first 2 weeks of the intervention period and around 1 h/week during weeks 3–10. Participants spent very few hours logged in (median 0) in weeks 11–16. Overall, participants completed a median (IQR) of 9 (2.8) modules during the 16‐week intervention period. Participants completed more modules in the early stages of the treatment period (weeks 0–3) and fewer new modules in later weeks (weeks 10–16). There was variation across participants in the number of modules completed: some completed a small number of modules in the early weeks of treatment but none thereafter; others completed modules throughout the 16 weeks of treatment. The number of participants receiving and completing each module is summarised in Table S2.

#### Therapist support

Overall, participants spent around 30 min on calls with their therapist during each week of the intervention period (median (IQR) = 33 (4.4)). Therapists made or received a median (IQR) of 21.0 (2.2) calls over the 16‐week intervention period. Almost all calls were with the young person rather than with their carer. More calls were made in the initial weeks (weeks 1–4; median = 2) and the last week (week 16; median = 2) of the treatment period, compared to weeks 5–15. Participants missed a mean of 17.6% of all offered phone appointments. Overall, young people sent and received around two text messages or emails per week during the 16‐week intervention period, and this number was relatively consistent across the intervention period.

#### Retention in treatment

All participants allocated to iCT‐PTSD‐YP started treatment, and none dropped out. Some participants continued with iCT‐PTSD‐YP after their 16‐week post‐treatment assessment. By their follow‐up assessment at week 38, participants had completed a median of 24.2 (IQR: 13.6) weeks of treatment.

### Clinical outcomes

#### Primary clinical outcome

The primary clinical outcome was the presence of PTSD at 16 weeks post‐randomisation, ascertained using the CAPS‐CA‐5. In iCT‐PTSD‐YP, 10/16 (62.5%) participants met PTSD caseness at 16 weeks compared to 11/13 (84.6%) in the WL arm. The iCT‐PTSD‐YP versus WL treatment effect odds ratio under ITT principles and adjusting for minimisation factors was 0.20 (95% CI: 0.02, 1.42), indicating that the odds of meeting PTSD caseness in the iCT‐PTSD‐YP arm were 80% lower than in the Wait List arm. However, the 95% confidence interval was wide and included the null value of 1. Two planned per‐protocol analyses were conducted, with some indication of a larger treatment effect in subpopulations who received essential core procedures (see Appendix S2).

#### Secondary clinical outcomes

Descriptive summary statistics and estimates of clinical effects for all secondary clinical outcomes are presented in Table 2.

**Table 2:** Secondary clinical outcomes

	iCT‐PTSD‐YP			Wait list			Adjusted mean difference^a^	Effect size^b^
	*n* = 16			*N* = 15				
	*M*	*SD*	*n*	*M*	*SD*	*n*		
							[95% CI]	[95% CI]
PTSD severity (CAPS‐CA‐5)								
Pre	42.3	9.1	16	40.9	9.1	15		
Post	27.1	14.1	16	36.9	9.8	13	−11.5	−1.2
							[−18.2, −3.9]	[−2.0, −0.4]
PTSD severity (CPSS‐5)								
Pre	52.9	12.5	16	50.1	13.2	15		
Post	32.4	20.1	16	43.2	12.5	13	−13.3	−1.0
							[−26.0, −0.5]	[−2.0, 0.0]
Follow up	28.3	20.8	13					
PTSD severity (CRIES)								
Pre	32.5	6.0	16	33.1	7.4	15		
Post	16.9	13.4	16	29.8	8.7	13	−13.4	−2.0
							[−20.0, −7.1]	[−3.0, −1.1]
Follow up	11.2	12.2	13					
Depression/anxiety (RCADS‐C)								
Pre	88.5	19.8	16	81.3	32.1	15		
Post	63.0	35.2	15	75.8	27.2	13	−26.7	−1.0
							[−45.4, −8.3]	[−1.7, −0.3]
Follow up	56.9	36.0	13					
Depression/anxiety (RCADS‐P)								
Pre	69.9	25.3	16	64.1	44.2	12		
Post	53.8	23.4	13	70.1	43.8	10	−23.1	−0.6
							[−42.3, −4.5]	[−1.2, −0.1]
Follow up	57.1	22.9	14					
Emotions and conduct (SDQ‐P)								
Pre	22.3	6.6	16	17.6	8.6	12		
Post	20.8	7.2	13	20.7	9.8	10	−6.7	−0.8
							[−11.4, −2.2]	[−1.4, −0.3]
Follow up	21.9	8.0	14					

^a^ Treatment effects were analysed using linear regression models where the dependent variable is the 16‐week outcome and covariates include treatment allocation, baseline scores of the respective measures, and all minimisation covariates. We report the adjusted mean differences given by the β coefficient for treatment allocation.

^b^ Effect size is Cohen's *d* calculated using the standard deviation of the baseline score for the respective measure.

#### Initial estimates of treatment effects

**Figure 2:**
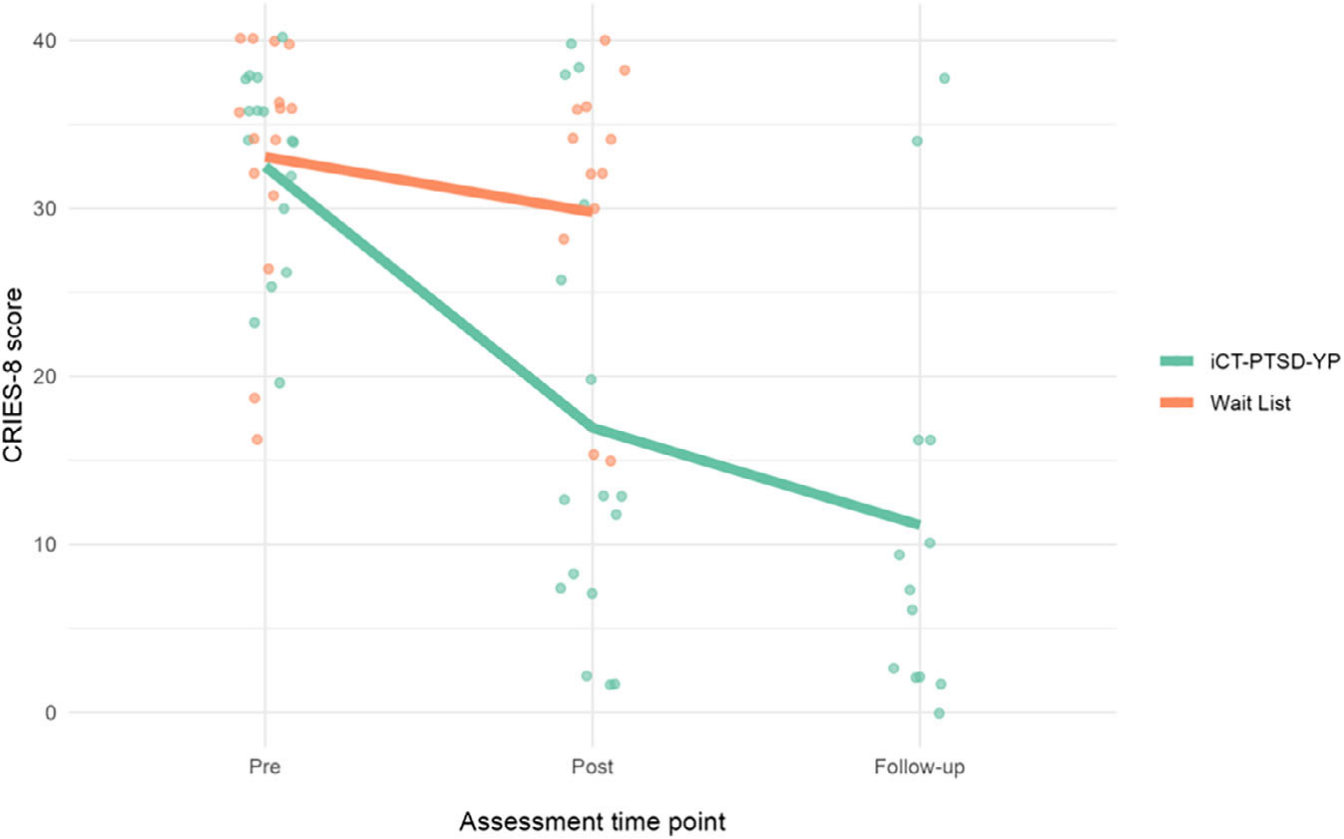
PTSD symptom severity CRIES‐8. This plot presents the observed scores on CRIES‐8 at pre‐intervention (baseline; 0 weeks), post‐intervention (16 weeks), and follow‐up (38 weeks, for participants in the iCT‐PTSD‐YP arm only). The lines represent the mean values by treatment allocation

At 16 weeks, the direction of all treatment effects indicates that, on average, participants allocated to iCT‐PTSD‐YP had better outcomes than those allocated to WL after adjusting for the baseline value of the outcome and the minimisation covariates. In all cases, the 95% confidence intervals excluded zero, suggesting initial evidence for the efficacy of iCT‐PTSD‐YP compared to WL. Between‐group effect sizes (Cohen's *d*) at 16 weeks were large to moderate, ranging from −2.0 (for CRIES‐8) to −0.6 (for RCADS‐P). At 38 weeks, further within‐group reductions in symptom severity were observed for all measures completed by young people in iCT‐PTSD‐YP (Table 2 and Figure 2).

#### Reliable improvement and reliable deterioration

At 16 weeks, 11/16 (69%) participants in iCT‐PTSD‐YP achieved reliable improvement from baseline on the CRIES‐8, compared to 1/13 (8%) in WL. At the same time point, 8/16 (50%) in iCT‐PTSD‐YP achieved reliable improvement on CPSS‐5, compared to 2/13 (15%) in WL. No young person in either arm showed reliable deterioration at 16 weeks. At 38‐week follow‐up (iCT‐PTSD‐YP arm only), 11/13 (85%) and 9/13 (69%) achieved reliable improvement on the CRIES‐8 and CPSS‐5, respectively. See Table S3.

#### Adverse events

There were no serious adverse reactions in either arm. There were two serious adverse events in iCT‐PTSD‐YP, neither related to study participation. In iCT‐PTSD‐YP, there were 7 adverse reactions, 6 of which comprised a temporary increase in PTSD symptoms after working on memory‐focused therapy modules. We recorded 32 adverse events in iCT‐PTSD‐YP and 16 in WL.

### Progression criteria

All four pre‐defined progression criteria were met: (1) 89% of eligible participants consented to participate; (2) 100% of randomised participants provided outcome data on a PTSD measure; (3) 94% of randomised participants completed at least one PTSD outcome at 16 weeks; and (4) Cohen's *d* for the CPSS at 16 weeks was −1.0 (95% CI: −2.0, 0.0).

### Exploratory mediation analysis

Participants allocated to iCT‐PTSD‐YP had lower scores on the CPTCI measure of appraisals at 6 weeks compared to WL, but this difference did not reach statistical significance (β (95% CI) = −2.88 (−7.44, 1.68)), and we found no evidence of between‐arm differences for other potential mediators. Higher scores on all three mediators at 6 weeks were associated with increased odds of meeting PTSD caseness at 16 weeks (Odds Ratios (OR) for appraisals (CPTCI) = 1.18 (0.95, 1.59); for rumination = 2.41 (1.08, 7.81); for memory quality (TMQQ) = 2.06 (1.17, 6.24)). The proportion of the total treatment effect mediated by appraisals was 22%; the proportion mediated by rumination was 11%, and the proportion mediated by memory quality was 3%. Appendix S3 provides further details and mediation results for secondary outcomes.

## Discussion

To our knowledge, this is the first study to evaluate an internet‐delivered therapy to treat young people with a primary diagnosis of PTSD. All pre‐specified feasibility and clinical thresholds for progression to a larger trial were met, indicating that a future scaled‐up confirmatory trial is warranted.

### Feasibility

Trial recruitment opened at the start of the COVID‐19 pandemic in the UK, which disrupted planned referral pathways, especially from schools. Recruitment rates may be higher in future trials if a school‐based referral pathway is open. Around half of assessed young people were eligible to participate. A common reason for ineligibility was the presence of PTSD symptoms relating to multiple traumatic events. Face‐to‐face CT‐PTSD‐YP has been adapted for young people with symptoms relating to multiple events (Allen et al., 2021), and in principle, iCT‐PTSD‐YP is adaptable for this group. Ineligibility due to lack of access to a phone or computer was very rare, but equity of access to technology remains a key consideration in future evaluations. Participant retention and data completeness were excellent.

### Adherence

The low drop‐out rate in the current study is within the range of rates reported in previous studies of digital interventions for PTSS in youth (0%–77%; Schulte et al., 2024), and in line with the drop‐out rate from face‐to‐face CT‐PTSD‐YP (Smith et al., 2007) and the adult version of internet‐delivered cognitive therapy (Ehlers et al., 2023). Regular therapist support in the current study is likely to have helped participants to complete treatment. Participants spent around 16 h logged into the App, more than the typical number of therapy hours in face‐to‐face CT‐PTSD‐YP (Smith et al., 2010). Participants tended to log in more often, for longer periods, and completed more modules at the beginning of therapy. Adherence may be increased by developing App content specific to common trauma types. Engagement may be increased if some therapist contact is face‐to‐face: we will explore this blended approach in future work.

Therapists spent around 30 min a week per participant on phone or video calls. The focus of the calls depended on the stage of therapy and the modules released: therapists supported young people to complete the therapy components. Thirty minutes per week is less than half the time spent in typical face‐to‐face TF‐CBTs but remains substantial and is more time than generally required in digital interventions for other youth mental health problems (Hollis et al., 2017). This amount of therapist contact may limit future scale‐up. The therapist's role differed from that in face‐to‐face therapy, with more frequent but shorter contacts with participants and more asynchronous contacts (commenting on participants' text input and sending emails or texts). Further research is needed to explore therapists' experience of delivering online therapy to young people, how this alters the therapeutic relationship, and the implications for improving efficacy and implementation. The structured nature of iCT‐PTSD‐YP is likely to enhance therapist fidelity to treatment. The release of some optional modules was lower than expected (see Table S2).

### Clinical outcomes

The odds of meeting PTSD caseness were estimated to be 80% lower after iCT‐PTSD‐YP compared to WL. Treatment effect sizes for all secondary clinical outcomes reported by young people were large, and those reported by parents were moderate to large. The improvements in clinical outcomes appeared durable, with 85% of participants reporting reliable improvement on the CRIES‐8 at the 38‐week follow‐up.

The loss of PTSD diagnosis in both arms was lower than in previous WL‐controlled trials of face‐to‐face CT‐PTSD‐YP (Meiser‐Stedman et al., 2017; Smith et al., 2007). The relatively low reliability of CAPS‐CA‐5 in the current trial suggests that diagnostic outcomes should be interpreted cautiously. The odds ratio of meeting PTSD caseness in the current trial is similar to the odds ratio found in a trial of face‐to‐face CT‐PTSD (Meiser‐Stedman et al., 2017), but both trials are small, and comparisons are made cautiously.

Large to moderate between‐group differences on secondary outcomes and continued improvement on these outcomes after treatment at follow‐up are in line with previous findings from face‐to‐face trials with adolescents. Effect size estimates on continuous PTSS outcomes in the current study compare favourably to those in previous studies of digital interventions (Schulte et al., 2024), although comparisons are made very tentatively given the modest size of the current trial and the wide variation in study population, design, and size of previous trials. In contrast to most previous digital interventions for PTSD, iCT‐PTSD‐YP includes key trauma‐focused modules and is delivered with regular therapist support; both aspects may contribute to the encouraging clinical outcomes.

Exploratory mediation analysis suggested that iCT‐PTSD‐YP exerted its effect in part by helping participants to update problematic trauma‐related appraisals. Clinical outcomes might be improved if iCT‐PTSD‐YP also reduced ruminative thinking and improved trauma memory coherence to a greater extent, for example via greater use of the optional module about rumination.

As expected in a small‐scale trial, confidence intervals were wide for all outcomes. We will seek to understand variations in outcome through our qualitative work, including investigation of participants' preferences for online compared to face‐to‐face therapy.

### Limitations

This early‐phase trial was not powered to detect differences between arms. We originally planned a three‐arm trial including face‐to‐face therapy, but the third arm was dropped before the trial started due to COVID‐related restrictions on face‐to‐face meetings (Smith et al., 2022). We measured time logged on, but young people might not have been actively using the App when they were logged on. We did not measure App use by parents. Potential adverse effects were not measured using a validated psychometric instrument. All participants had developed PTSD following a single event trauma, and most participants were female and White, limiting generalisability.

### Clinical implications and future work

We found that iCT‐PTSD‐YP is broadly acceptable to young people with PTSD, requires approximately half the therapist time compared to face‐to‐face therapy, and has potential for meaningful and sustained clinical effects. The nature and intended use of existing digital interventions for trauma‐exposed youth vary enormously (Schulte et al., 2024), ranging from web‐based interventions implemented at scale in the community (Ruggiero et al., 2015) to gamified interventions delivered to youth with clinically relevant symptoms (Schuurmans et al., 2020). Current findings, if confirmed in a larger trial, suggest that there is also a role for clinic‐based implementation of therapist‐supported digital interventions for treatment‐seeking youth with a PTSD diagnosis, including those with severe PTSS. Future work is needed to refine the intervention and its delivery and to evaluate it rigorously in a scaled‐up trial.

## Ethical considerations

The trial was approved by a UK Health Research Authority (HRA) Research Ethics Committee (19/LO/1354).

## Trial registration

The trial was prospectively registered (including the study protocol and statistical analysis plan) with the ISRCTN (registry ID number 16876240; https://doi.org/10.1186/ISRCTN16876240).

**Key points**

- Highly effective psychological therapies for treatment of PTSD exist, but most adolescents with PTSD do not receive effective evidence‐based therapy.
- Digital technology has the potential to widen the availability of effective therapy.
- We developed a novel smartphone App and website to deliver Cognitive Therapy for PTSD in young people via the internet (iCT‐PTSD‐YP).
- In an early‐stage trial, we found that iCT‐PTSD‐YP, delivered with therapist support, was acceptable to young people and showed promising clinical effects.
- A future confirmatory trial is warranted and appears feasible to run.

## Supporting information


**Appendix S1.** Development process.
**Appendix S2.** Per protocol analyses.
**Appendix S3.** Exploratory mediation analysis.
**Figure S1.** Detailed CONSORT diagram.
**Table S1.** Assessment schedule.
**Table S2.** Module completion.
**Table S3.** Reliable improvement.

## Data Availability

The authors' intentions are to maximise the availability and sharing of their data for the benefit of the wider research community while providing for its long‐term preservation. Data released to the wider community after publication will be fully anonymised. Upon reasonable request from an external researcher, the Project Management Group will make the decision on whether to supply anonymised research data.
