# Supplementary material for: Early‐stage randomised controlled trial of therapist‐supported online cognitive therapy for post‐traumatic stress disorder in young people
Source: J Child Psychol Psychiatry. 2025 Feb 6;66(8):1117–28. doi: 10.1111/jcpp.14124 (PMC12267680; doi:10.1111/jcpp.14124)
Supplement: Supplementary file 1 — Appendix S1. Development process. Appendix S2. Per protocol analyses. Appendix S3. Exploratory mediation analysis. Figure S1. Detailed CONSORT diagram. Table S1. Assessment schedule. Table S2. Module completion. Table S3. Reliable improvement. [file JCPP-66-1117-s001.docx]

**Supplementary Table 1**: assessment schedule

| MEASURE | STUDY PERIOD | | | | | |
| --- | --- | --- | --- | --- | --- | --- |
|  | **Screen**  0-1 weeks | **Pre**  0 weeks | **Weekly**  (*iCT only*) | **Mid**  0+ 6 weeks | **Post**  0+ 16 weeks | **Follow-up**  0+38 weeks (*iCT only*) |
|  |  |  |  |  |  |  |
| ENROLMENT |  |  |  |  |  |  |
| Eligibility screen | x |  |  |  |  |  |
| *Provide study information* | *x* |  |  |  |  |  |
| *Gain informed consent* |  | *x* |  |  |  |  |
| *ONLINE ASSESSMENT*  *DAWBA* |  | *x* |  |  |  |  |
|  |  |  |  |  |  |  |
| *INTERVIEW* |  |  |  |  |  |  |
| *DEMOGRAPHIC INTERVIEW*  *CAPS-CA-5* |  | *x*  *x* |  |  | *x* |  |
| *CGAS* |  | *x* |  |  | *x* |  |
|  |  |  |  |  |  |  |
| *ADOLESCENT QUESTIONNAIRES* |  |  |  |  |  |  |
| *CPSS-5* |  | *x* |  |  | *x* | *x* |
| *CRIES-8* |  | *x* | *x* | *x* | *x* | *x* |
| *RCADS-C* |  | *x* |  |  | *x* | *x* |
| *CPTCI* |  | *x* |  | *x* | *x* | *x* |
| *TMQQ* |  | *x* |  | *x* | *x* | *x* |
| *Rumination items^1^* |  | *x* |  | *x* | *x* | *x* |
| *CHU-9D* |  | *x* |  |  | *x* | *x* |
| *Adverse events* |  |  |  | *x* | *x* | *x* |
|  |  |  |  |  |  |  |
| *CARER QUESTIONNAIRES* |  |  |  |  |  |  |
| *SDQ-P* |  | *x* |  |  | *x* | *x* |
| *RCADS-P* |  | *x* |  |  | *x* | *x* |
| *CA-SUS* |  | *x* |  |  | *x* | *x* |
| *Adverse events* |  |  |  | *x* | *x* | *x* |
|  |  |  |  |  |  |  |

DAWBA: Development and Wellbeing Assessment, CAPS-CA-5: Clinician Administered PTSD Scale for DSM-5, Child and Adolescent version; CGAS: Children’s Global Assessment Scale; CPSS-5: Child PTSD Symptom Scale for DSM-5; CRIES-8: Children’s Revised Impact of Event Scale; RCADS: Revised Children’s Anxiety and Depression Scale; CPTCI: Child Post Traumatic Cognitions Inventory; TMQQ: Trauma Memory Quality Questionnaire; CHU-9: Child Health Utility Index; SDQ-P: Strength and Difficulties Questionnaire; CASUS: Child and Adolescent Service Use Schedule

1. Three items from the Trauma Related Rumination Questionnaire (Meiser-Stedman et al 2014): *I keep wishing that I could go back in time and prevent the event from happening; whenever I think about the event I wonder why it happened to me; I am always wondering if my family or I might get hurt again*

**Appendix S1:**

Internet-delivered Cognitive Therapy for PTSD in young people (iCT-PTSD-YP)

Development process

iCT-PTSD-YP was built by a commercial company under contract with King’s College London. We ran workshops with the designers to explain therapy principles and components so that a wireframe for the intervention could be developed. We ran schools-based focus groups with young people in the target age range, where we presented mood boards of design options to help explore their views on overall visual language; and we asked them about device usage and preferences for Apps or browsers. Wireframes and visual designs (informed by the focus groups) were combined to produce prototype modules. Individual prototypes were tested by young people who were receiving face to face CT-PTSD-YP in our clinic. Each prototype was revised as needed based on young people’s feedback, and then re-tested by them. Iterative testing and development of prototype modules resulted in a comprehensive working App for young people comprising all core and optional modules (see below). We then employed this App in an uncontrolled development case series (N=11) to gather feedback on adherence, technical functioning, acceptability (design appeal, ease of use, comprehension), safety, and delivery. Final changes to the App were made during the case series based on this feedback. The App is built as a Progressive Web App (PWA) so that it can be used seamlessly across devices including phones (where it appears to function as an App), and laptop or desktop computers (where it appears as a website).

iCT-PTSD-YP content

The intervention is based on a theoretical model of PTSD developed by Ehlers and Clark (2000) and adapted by Meiser-Stedman (2002) for use with children and young people. The intervention includes all components from our published manual of face-to-face CT-PTSD for young people (Smith et al 2010). Module content was informed by iCT-PTSD for adults (Ehlers et al 2023). We developed 11 core modules intended to be used by all participants; plus 11 additional optional modules to be used depending on the cognitive formulation (appraisals linked to certain emotions, maintaining cognitive strategies) and comorbidity.

| **Core modules** | **Optional modules** |
| --- | --- |
| What is PTSD | Guilt |
| Reclaiming life | Shame |
| It’s understandable | Anger |
| Your story | Physical Difference |
| Hotspots | Rumination |
| Updating your story | Self-critical thinking |
| Triggers | Images |
| Sense of danger | Grief |
| Viewing the site virtually | Panic |
| Viewing the site in person | Sleep |
| Blueprint | Relaxation |

Each module includes: brief ratings of mood and posttraumatic stress symptoms entered by the user; psychoeducation about the topic of the module, presented via text and narrated animations; examples of young people with similar problems employing the treatment component, presented via three “audio stories” of prototypical case examples; personalised user input about the treatment component (eg, scheduling activities in “reclaiming life”; writing about the traumatic event in “your story”; identifying problematic cognitions in “hotspots”); and short “video testimonies” of a young person talking about their experience of CT-PTSD.

App use is intended to be individualised according to the young person’s presenting problems and motivation. Modules are released to the young person by the therapist as required and can be accessed by the young person for self-study at any time once released. Modules can be completed in one long sitting, or over several shorter sittings. The amount of time to be spent on each module was not prescribed. As with face-to-face CT-PTSD-YP, the sequence of core treatment components generally follows the order in the table above, but optional modules may be released early if judged by the therapist to be helpful.

Therapist support

The App is used with therapist support. Therapists use clinical judgement to decide the order and pace of module release. Therapists can view user input to the App: for example, they can see the young person’s symptom ratings, their activity plan, their trauma narrative, and their hotspots. Therapists can add input that is viewable by the user: for example, they can make suggestions or comments on the user’s trauma narrative. Therapists contact young people via videocall, phone call, or the within-App messaging function. No limits were set on message content. We planned for around 15 minutes per week of remote therapist contact by phone or videocall, but we did not set limits on the amount of therapist contact or messages.

Carer App

We developed a parallel App for carers of young people with PTSD. The purpose of the carer App was to provide information about the treatment rationale and components. Information is provided via text, and by re-using some of the animations developed for young people. Each carer module included a section on common questions or concerns, with answers, advice, and suggestions. Carers were not prompted to input any information into the App. Carer modules were not released sequentially: when parents logged into the App, they could view all modules. Module release for carers was not personalised: all carers accessed the same modules.

Carer modules:

What is PTSD

Reclaiming life

It’s understandable

Your child’s story

Hotspots and updating

Triggers and sense of danger

Site visits

Looking to the future

**Appendix S2**

Per protocol analyses

In these secondary analyses, we sought to estimate the treatment effect among participants who had completed the minimum therapy needed to achieve benefit, based on the cognitive model of PTSD (Ehlers and Clark, 2000).

We planned and conducted two per-protocol analyses.

In the first per-protocol analysis, participants were considered to have met the definition of completing the minimum therapy needed to achieve benefit if they completed the following six (of 11) core modules: *What is PTSD, Reclaiming Life, It’s Understandable; Your story; Hotspots; and Updating Your Story.* Under this definition (N=26), the treatment effect odds ratio (N=26) was 0.15 (95% CI: 0.01, 1.15).

In the second per protocol analysis, in addition to meeting the criteria above, participants also completed the module on *Working with Triggers.* Under this definition (N=25), the treatment effect odds ratio was 0.14 (95% CI: 0.01, 1.12).

**Appendix S3**

Exploratory mediation analysis

**Methods**

We carried out an exploratory analysis to assess how much of the effect of the iCT-PTSD-YP versus Wait List (WL) on the primary and selected secondary clinical outcomes was conveyed via three potential mediators:

1. Child Post Traumatic Cognitions Inventory score (CPTCI);
2. Trauma Memory Questionnaire (TMQQ); and
3. Trauma-related rumination items.

Potential mediators were measured during treatment (6 weeks post-randomisation).

We pre-specified an exploratory mediation analysis for the primary clinical outcome in our protocol and statistical analysis plan. However, in this supplement, we additionally report mediation involving three of the secondary clinical outcomes: the CAPS-CA-5 total score, the CPSS-5 total score, and the CRIES-8. These secondary mediation analyses were not pre-specified but may be of interest to readers.


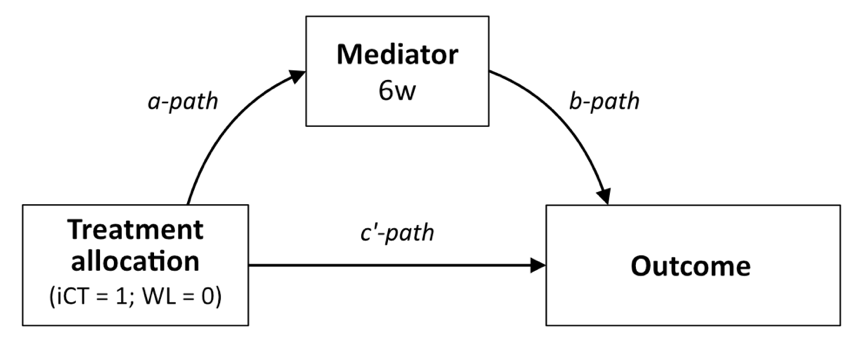


**Figure A** – Mediation model

We conducted the mediation analyses in two parts.

*Estimation of separate a- and b-paths*

First, we used separate regression models to estimate the effect of treatment allocation on each potential mediator at 6 weeks (*a-path*), the effect of each potential mediator on each clinical outcome at 16 weeks (*b-path*), and the total effect of treatment allocation on each outcome (*c-path*).

For the primary clinical outcome (presence of PTSD at 16 weeks), we used separate linear regression models to estimate the *a-paths* and separate binary logistic regression models to estimate *b-paths* and *c-paths*. For secondary clinical outcomes (continuous), we used separate linear regression models to estimate the *a-*, *b-*, and *c-paths*.

All models were adjusted for the minimisation covariates of sex (0 = Male; 1 = Female) and PTSD severity at baseline (0 = Low; 1 = High). Linear regression models for the secondary clinical outcomes (*b-* and *c-paths*) additionally included the baseline measure of the respective outcome. All models were estimated in R using the lm and glm functions for linear and binary logistic models, respectively.

*Estimation of* *natural indirect effects*

Second, we calculated the indirect effects for each mediator and outcome using the regression-based method of Valeri and VanderWeele (2013), implemented in the regmedint package for R (Yoshida, 2022). This approach decomposed the total effect (TE) into the pure natural direct effect (PNDE) and the total natural indirect effect (TNIE), following Pearl (2001):

$$\begin{matrix} PNDE & =E\left[ Y_{a_{1},M_{a_{0}}}|C=c \right]-E\left[ Y_{a_{0},M_{a_{0}}}|C=c \right] \\ TNIE & =E\left[ Y_{a_{1},M_{a_{1}}}|C=c \right]-E\left[ Y_{a_{1},M_{a_{0}}}|C=c \right] \end{matrix}$$

For the primary clinical outcome, we followed the recommendation of Valeri and VanderWeele to use a log-linear model for the outcome model, given the common binary outcome. This was implemented as a modified Poisson model (log link with robust variance; Zou, 2004), with the effects given on the risk ratio scale. The approximate proportion of the total effect mediated via each mediator was calculated on the risk ratio scale, following VanderWeele (2015; p. 48). For the secondary clinical outcomes (continuous), we used a linear regression model for the outcome. For all outcomes, confidence intervals for the PDNE and TNIE were calculated using a percentile bootstrap with 1000 replications. We assumed no treatment-mediator interaction.

This analysis included participants in the modified ITT sample (all participants with a non-missing outcome according to the treatment to which they were randomised) who also provided information on all three potential mediators.

**Results**

The analytical sample for mediation analyses included 26/29 participants (14 from iCT; 12 from Wait List) from the modified ITT sample with complete information for the three potential mediators, the primary outcome, and the selected secondary outcomes.

*Primary outcome*

| 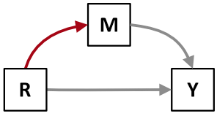 | For the *a*-path, participants allocated to iCT-PTSD-YP had lower scores on the CPTCI measure of appraisals (β = -2.88 (-7.44, 1.68)) than those allocated to WL, but this was not statistically significant. There was no evidence of a between-arm difference for the other potential mediators at 6 weeks, for which differences centred on zero with wide confidence intervals (Rumination β = -0.29 (-1.59, 1.01); TMQQ β = -0.20 (-3.37, 3.06)).  This suggests that at 6 weeks post-randomisation, the treatment only affected the CPTCI mediator. One implication of this for the future is that the treatment and/or its delivery may need to be refined to affect the other putative mediators if they remain important theoretical treatment targets. However, as shown in Supplementary Table 2, no participants completed the rumination module. This may explain the lack of between-arm differences in the rumination mediator and suggests it would be helpful for therapists to check for the presence of rumination and release the rumination module if needed. Another possibility is that change in the mediators may have occurred later than 6 weeks, suggesting the need for later assessments of these variables. |
| --- | --- |
| *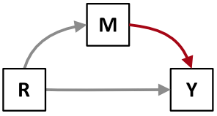* | For the *b*-path, higher scores on all three potential mediators led to increased odds of meeting PTSD caseness at 16 weeks (Odds Ratios (OR) for appraisals (CPTCI) = 1.18 (0.95, 1.59); for rumination = 2.41 (1.08, 7.81); for memory quality (TMQQ) = 2.06 (1.17, 6.24)).  This suggests that all three of the mediators were useful targets for the treatment because all three had a positive effect on the outcome, indicating that worse (i.e., increased) scores on each mediator lead to increased odds of PTSD caseness. There were larger effects for the trauma-related rumination items and TMQQ, compared to CPTCI. |
| *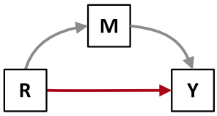* | For the *c*-path, the total effect of treatment allocation on the primary outcome was consistent with results presented in the main text (OR (95% CI) = 0.15 (0.01, 1.23)). |

Consistent with these path estimates, the proportion of the total treatment effect on the primary outcome mediated by appraisals was calculated as 22%, whereas the proportion mediated by rumination was 11%, and the proportion mediated by memory quality was 3%. This provides some support for CPTCI as a mediator but weak or no support for other potential mediators.

*Secondary outcomes*

| 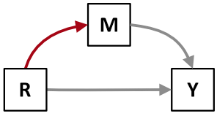 | See above for *a*-path results (these are unchanged for different outcomes). |
| --- | --- |
| *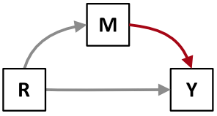* | For the *b*-path, the results for secondary outcomes are more mixed. All estimated coefficients were positive, indicating that higher scores on a given mediator were associated with higher scores on the outcome. However, the estimated effects were small, and few reached statistical significance.  The β coefficients (and 95% confidence intervals) for each mediator and outcome are presented below:   \|  \| Appraisals  (CPTCI) \| Rumination \| Memory quality  (TMQQ) \| \| --- \| --- \| --- \| --- \| \| CAPS-CA-5 total score \| 0.58  (-0.24, 1.39) \| 3.20  (0.42, 5.98) \| 0.98  (-0.28, 2.25) \| \| CPSS-5 total score \| 0.85  (-0.47, 2.16) \| 4.35  (-0.03, 8.73) \| 2.27  (0.63, 3.92) \| \| CRIES-8 \| 0.30  (-0.47, 1.06) \| 1.15  (-1.60, 3.90) \| 0.62  (-0.48, 1.171) \|   As above, this suggests that the chosen mediators were useful targets for the treatment because all three were positively associated with the secondary outcomes, indicating that worse (i.e., increased) scores on each mediator lead to worse outcomes (i.e., increased scores) for each outcome. |
| *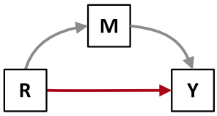* | For the *c*-path, the total effects of treatment allocation on the secondary outcomes were consistent with the results presented in the main text. |

Consistent with the *b*-path results, above, the proportion of the total effect on the secondary outcomes mediated by the potential mediators was low, ranging from 0% to 16%. For CAPS-CA5 total score, the proportion mediated was 16% (CPTCI), 13% (rumination), and 7% (TMQQ). For CPSS-5 total score the proportion mediated was 15% (CPTCI), 9% (rumination), and 6% (TMQQ). For CRIES-8, the proportion mediated was 5% (CPTCI), 2% (rumination), and 0% (TMQQ). Note that each mediator was tested in a separate model.

**Summary**

This exploratory mediation analysis found preliminary support for the role of CPCTI as a mediator of the treatment effect (allocation to iCT) on the primary and secondary clinical outcomes. However, the analysis was underpowered, and most effects did not reach statistical significance. We found little support for the role of other mediators (rumination items and memory quality (TMQQ)), most likely due to the absence of the a-path; i.e., the iCT intervention did not affect scores for these mediators when measured at 6 weeks.

This suggests that the treatment and/or its delivery would need to be refined to have effects on these treatment targets. The *b*-path results suggest if the treatment and/or its delivery could be refined to have an effect on rumination and TMQQ (and possibly more of an effect on CPTCI), there would be subsequent positive effects on the outcome.

**References**

Pearl J. Direct and indirect effects. In: *Proceedings of the Seventeenth conference on Uncertainty in artificial intelligence*. San Francisco, CA, USA: Morgan Kaufmann Publishers Inc. 2001. 411–20.

Valeri L, VanderWeele TJ. Mediation analysis allowing for exposure–mediator interactions and causal interpretation: Theoretical assumptions and implementation with SAS and SPSS macros. *Psychological Methods* 2013;**18**:137–50. doi:[10.1037/a0031034](https://doi.org/10.1037/a0031034)

VanderWeele T. *Explanation in Causal Inference: Methods for Mediation and Interaction*. Oxford University Press 2015.

Yoshida K, Li Y. *Regmedint: Regression-based causal mediation analysis with interaction and effect modification terms*. 2022. <https://kaz-yos.github.io/regmedint/>

Zou G. A Modified Poisson Regression Approach to Prospective Studies with Binary Data. *American Journal of Epidemiology* 2004;**159**:702–6. doi:[10.1093/aje/kwh090](https://doi.org/10.1093/aje/kwh090)

**Supplementary Figure 1:**

Detailed CONSORT diagram


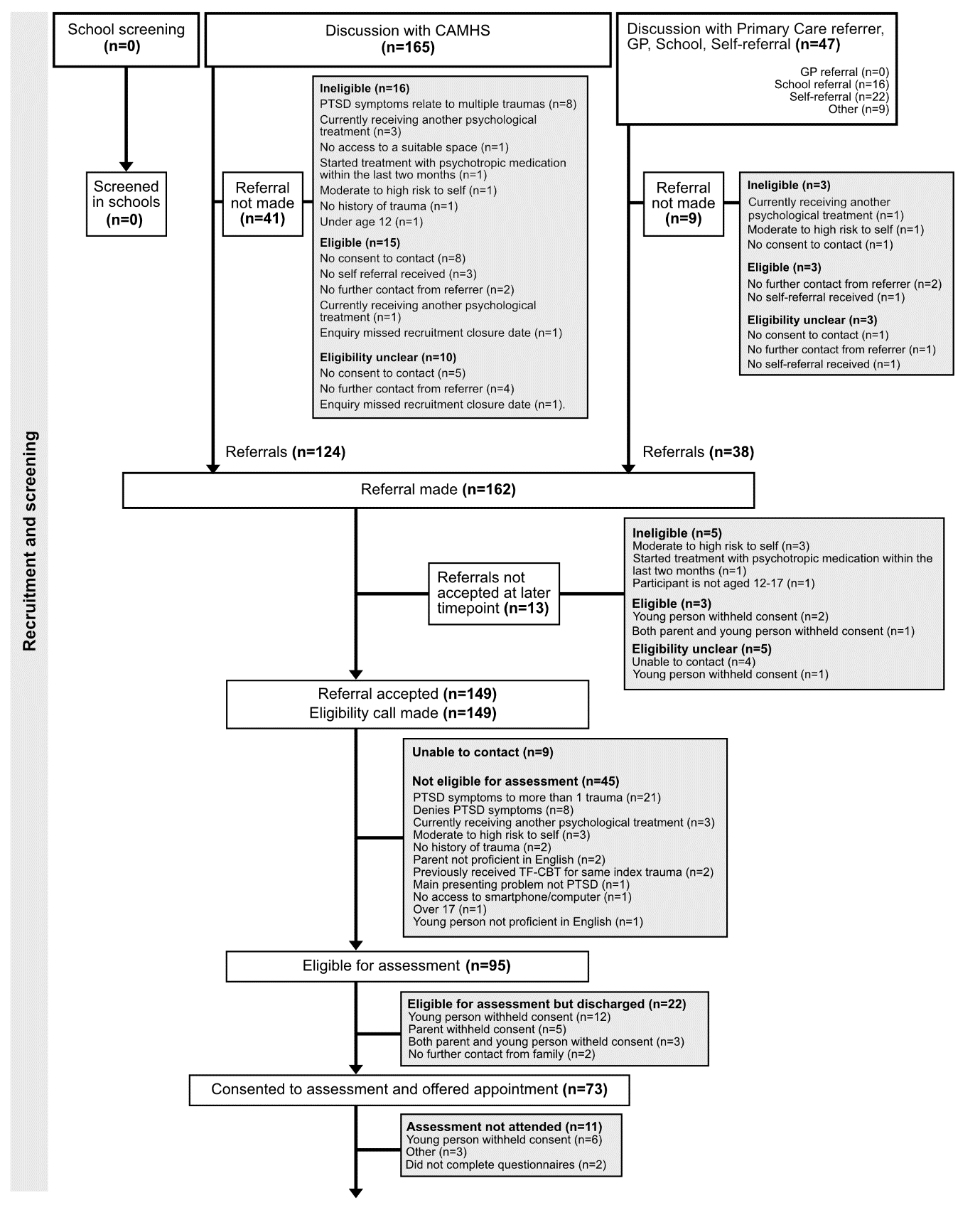


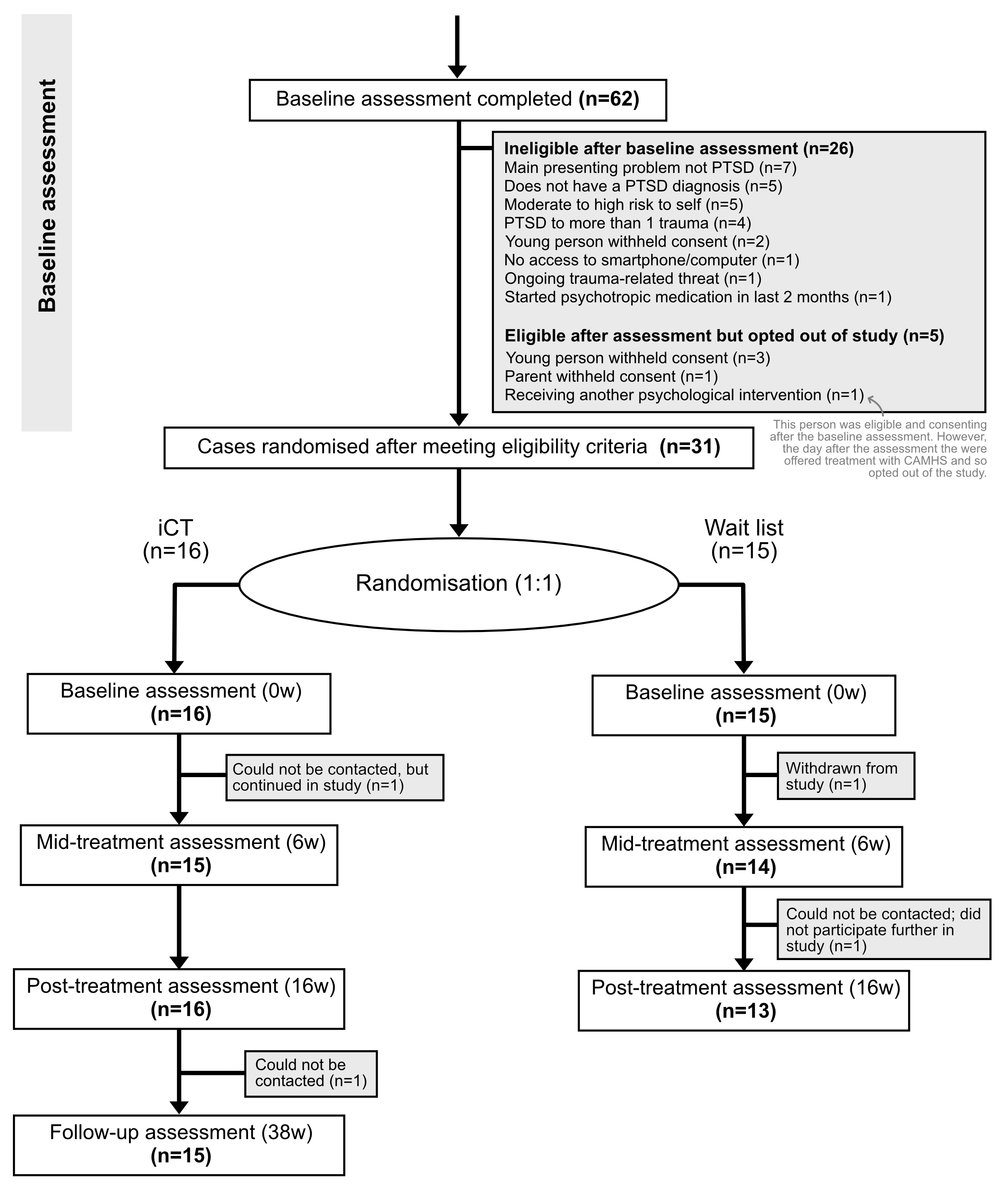


Supplementary Table 2: Module completion

| Core  Module | Number of participants^1^ | Proportion of participants**^2^** | Optional Module | Number of participants^1^ | Proportion of participants^2^ |
| --- | --- | --- | --- | --- | --- |
|  |  |  |  |  |  |
| What is PTSD | 16 | 100%  16/16 | Guilt | 9 | 100%  9/9 |
| Reclaiming life | 16 | 100%  16/16 | Images | 5 | 83%  5/6 |
| It’s understandable | 16 | 100%  16/16 | Relaxation | 2 | 50%  2/4 |
| Your story | 16 | 100%  16/16 | Self-critical thinking | 1 | 100%  1/1 |
| Hotspots | 14 | 100%  14/14 | Grief | 1 | 100%  1/1 |
| Updating your story | 13 | 93%  13/14 | Sleep | 0 | N/A  0/0 |
| Triggers | 13 | 93%  13/14 | Anger | 0 | 0%  0/1 |
| Sense of danger | 9 | 100%  9/9 | Shame | 0 | N/A  0/0 |
| Viewing the site virtually | 4 | 100%  4/4 | Rumination | 0 | N/A  0/0 |
| Viewing the site in person | 7 | 100%  7/7 | Panic | 0 | N/A  0/0 |
| Blueprint | 11 | 92%  11/12 | Physical difference | 0 | N/A  0/0 |
|  |  |  |  |  |  |

1: Total number of participants who completed the module

2: Number who completed the module as a proportion of those to whom the module was released

Supplementary Table 3:

Number (%) achieving reliable improvement or deterioration for secondary outcomes CPSS-5 and CRIES-8.

|  | **Mid-treatment (6w)** | | **Post-treatment (16w)** | | **Follow-up (38w)** | |
| --- | --- | --- | --- | --- | --- | --- |
|  | **Wait List** | **iCT** | **Wait List** | **iCT** | **iCT** |  |
| **CRIES-8** | | | | | | |
| No change | 13/14 (93) | 12/15 (80) | 12/13 (92) | 5/16 (31) | 2/13 (15) |  |
| Reliable deterioration | 1/14 (7) | 0/15 (0) | 0/13 (0) | 0/16 (0) | — |  |
| Reliable improvement | 0/14 (0) | 3/15 (20) | 1/13 (8) | 11/16 (69) | 11/13 (85) |  |
| **Child Post Traumatic Stress Scale (CPSS-5)** | | | | | | |
| No change | — | — | 11/13 (85) | 8/16 (50) | 4/13 (31) |  |
| Reliable deterioration | — | — | 0/13 (0) | 0/16 (0) | — |  |
| Reliable improvement | — | — | 2/13 (15) | 8/16 (50) | 9/13 (69) |  |
